# Importance of HPV in Chinese Penile Cancer: A Contemporary Multicenter Study

**DOI:** 10.3389/fonc.2020.01521

**Published:** 2020-09-04

**Authors:** Weijie Gu, Peipei Zhang, Guiming Zhang, Jiaquan Zhou, Xuefei Ding, Qifeng Wang, Beihe Wang, Yu Wei, Shengming Jin, Dingwei Ye, Yao Zhu

**Affiliations:** ^1^Department of Urology, Fudan University Shanghai Cancer Center, Shanghai, China; ^2^Department of Oncology, Shanghai Medical College, Fudan University, Shanghai, China; ^3^Department of Pathology, Ruijin Hosiptial, Jiaotong University, Shanghai, China; ^4^Department of Urology, The Affiliated Hospital of Qingdao University, Qingdao, China; ^5^Department of Urology, Hainan General Hospital, Haikou, China; ^6^Department of Urology, Northern Jiangsu People's Hospital, Yangzhou, China; ^7^Department of Pathology, Fudan University Shanghai Cancer Center, Shanghai, China; ^8^Department of Pathology, Affiliated Hospital of Jiangnan University, Wuxi, China

**Keywords:** penile cancer, human papillomavirus, prevalence, genotype, China

## Abstract

**Objective:** To investigate the HPV DNA prevalence and genotype distribution among penile cancer in China. To identify association between HPV prevalence, different histological subtypes, tumor stage, tumor grade, demographics, comorbidity, and phimosis incidence trend. Standardized HPV DNA detection and p16^*INK4a*^ expression were used in a multi-center series of 340 penile squamous cell carcinomas diagnosed from 2006 to 2017.

**Materials and Methods:** HPV DNA detection and genotyping were examined by a validated kit for 23 different HPV subtypes (PCR-RDB HPV test). The cases with positive HPV DNA were additional tested for p16^INK4a^ expression to confirm the HPV infection.

**Results:** Using the PCR-RDB HPV test, overall HPV prevalence was 48.8% (166/340) and that of p16^INK4a^ expression was 45.6%. In this studied population, HPV16 was the most frequent HPV type detected in HPV-positive cancers (76.5%). HPV18 was the second most common type in penile cancers (15.1%). After pathology review, 307 cases were confirmed as invasive penile cancer, and the other 33 were non-invasive caners. The histologic subtypes of warty, basaloid, clear cell papillary, adenosquamaous and pseudohyperplastic were showed high HPV DNA prevalence. Among invasive cancers, no statistically significant differences in prevalence were observed by tumor grade, tumor stage or lymphnode stage at diagnosis. HPV positive penile cancer incidence significantly increase and the phimosis incidence significantly decrease from 2006 to 2017.

**Conclusions:** About a half of penile cancers were related to HPV infection. Our findings highlight the phimosis related penile cancers have been declining, the HPV related in the development of penile cancer and a fully aware of regional differences in HPV genotype distribution are tasks for penile cancer control and prevention.

## Introduction

Penile cancer is relatively rare in the developed nations of Western Europe and the United States, with an age-standardized incidence of 0.3–1.0 per 100,000 men (1). However, the prevalence of penile cancer in developing areas of Africa, Asia and South America ranges from 6 to 20 per 100,000 men (2). The etiology of penile cancer is multifactorial; it is linked to inflammation, smoking, phimosis, poor personal hygiene, and human papillomavirus (HPV) infection (3). HPV is regarded as the most frequently acquired sexually or not sexually transmitted disease, with more than 6 million new cases transmitted annually in the United States. In addition, HPV is implicated in both benign and malignant diseases, including cervical, head and neck, anal, and penile cancers. There is strong evidence linking the development of penile cancer to infection with high-risk HPV. A new World Health Organization (WHO) classification of penile cancer published in 2016 categorized penile cancer into HPV-related and non-HPV-related tumors (4). Basaloid and warty penile squamous cell carcinomas are considered HPV-related, while non-HPV-related tumors mainly include the usual type, papillary type and verrucous carcinoma.

Recently, Alemany et al. reported the largest epidemiologic study to evaluate the role of HPV infection in penile cancer, examining 1,010 penile cancer specimens from 25 countries (5). One-third to one-quarter of penile cancers were related to HPV infection. The high-risk oncogenic subtypes 16 and 18 accounted for the most frequent subtype, detected in over 70% of HPV-positive specimens. In that study, only 71 Asian samples (7%) were enrolled, and fewer cases in Asian patients (18%) were associated with HPV infection. To date, no substantial data have been reported, and the importance of HPV infection in China remains unclear. China has a huge population and correspondingly a large numbers of penile cancer patients. The best method for combating HPV-related disease is to prevent it with routine vaccination. Therefore, it is important to understand the exact HPV prevalence in China and the specific HPV genotype distribution.

We therefore sought to investigate the HPV DNA prevalence and genotype distribution among penile cancer cases in China. We also intend to identify association between HPV prevalence, different histological subtypes, tumor stage, tumor grade, demographics, comorbidity, and phimosis incidence trend. Standardized HPV DNA detection and p16^*INK4a*^ expression were used in a multicenter study of 340 penile squamous cell carcinomas.

## Materials and Methods

### Ethics Approval

Informed consent was obtained for all subjects. The patients enrolled signed consent before the treatment for future use the blood or tissue samples for scientific research. The protocols for this study were approved by the Institutional Review Board of Fudan University Shanghai Cancer Center, The Affiliated Hospital of Qingdao University, Hainan General Hospital, Northern Jiangsu People's Hospital, and Affiliated Hospital of Jiangnan University.

### Patient Samples and Clinical Data

A multicenter retrospective cross-sectional study was designed and coordinated by the Fudan University Shanghai Cancer Center. In this study, we used 340 formalin-fixed, paraffin-embedded (FFPE) penile cancer specimens diagnosed from 2006 to 2017 that were obtained from 5 level three tertiary hospitals in China (Fudan University Shanghai Cancer Center, The Affiliated Hospital of Qingdao University, Hainan General Hospital, Northern Jiangsu People's Hospital, and Affiliated Hospital of Jiangnan University). Patients with suspiciously invasive penile cancer underwent complete glansectomy, partial or total penectomy. Patients initially diagnosed with pT1b-pT4 or cN1 stage underwent inguinal lymphadenectomy. Patients with more than 2 inguinal lymphnode metastasis, Cloquet's inguinal metastasis, extranodal extension or radiologically suspicious pelvic lymphnode metastasis underwent pelvic dissection. Information about age and year of diagnosis and lymph node metastasis were also obtained from the participating centers. Comorbidities such as smoking, drinking, hypertension, and diabetes were also considered in this study.

### Histopathologic Evaluation

The first sections of the FFPE samples were used for histopathologic evaluation after hematoxylin and eosin (HE) staining. All cases were reviewed by a specialized pathologist (Peipei Zhang) from Fudan University Shanghai Cancer Center. The pathological evaluation was performed following the WHO criteria proposed in 2016 (6). All the patients were staged according to the latest published 8th edition American Joint Committee on Cancer TNM system.

Diagnosis included confirmation of penile squamous cell carcinoma, assessment of the subtypes, depth of invasion, lymph node metastasis, extranodal involvement, the proportion of tumor to the whole tissue section, and the adequacy of the sample for HPV testing.

Only the histological confirmation of primary squamous cell penile carcinoma cancer was included. For each tumor tissue sample, sections with the confirmed presence (>70%) of tumor cells were selected for further HPV DNA analysis.

Our pathologist finally confirmed 307 cases were invasive penile cancer. The other 33 were non-invasive caners, most of them were verrucous type (26/33), and 8 of them underwent inguinal lymphadenectomy.

### DNA Extraction and Polymerase Chain Reaction (PCR) Conditions

The paraffin samples were processed under strict conditions to avoid potential contamination as described in a previous study. DNA from paraffin-embedded tissue blocks was extracted from 4 to 5 sequential unstained sections, each 4-mm thick (7). Genomic DNA was extracted with the QIAamp DNA FFPE Tissue Kit (Qiagen) following the manufacturer's recommendations. The quality of the extracted DNA was verified using a spectrophotometer (260/280 nm ultraviolet light). The amplification was tested using the housekeeping gene GAPDH with the same reactions as an internal positive control to ensure the quality of the DNA. A verified HPV multiple infection cervical intraepithelial neoplasia sample was used as a positive control, and distilled water was used as a negative control. To avoid contamination within the lab, all specimens were independently tested in two isolated labs by blind assignment.

### HPV DNA Genotyping for the 23 Types

HPV DNA detection was performed using the Human Papillomavirus Genotyping assay for 23 genotypes from Yaneng BIOscience (ShenZhen Co., Ltd., China), which was approved by the China Food and Drug Administration for diagnosis. The system provides a reliable and sensitive clinical reference for HPV detection and has been widely used in cervical cancer screening in China (8). The system can identify 18 high-risk HPV types (16, 18, 31, 33, 35, 39, 45, 51, 52, 53, 56, 58, 59, 66, 68, 73, 82, and 83) and 5 low-risk HPV types (6, 11, 42, 43, and 81).

### p16^*INK*4*a*^ Expression

Immunohistochemical p16^*INK4a*^ expression was evaluated in all specimens. Overexpression of p16^*INK4a*^ can serve as a surrogate marker for transcriptionally active HPV infection due to its strong correlation with high-risk HPV genotype infection (9). p16^*INK4a*^ was detected using the p16^*INK4a*^ kit (Dako, Glostrup, Denmark) following the manufacturer's protocol. A pattern of diffuse nuclear and cytoplasmic staining of >70% of the tumor cells was considered positive.

### Statistical Analysis

The HPV DNA prevalence was calculated among HPV DNA-analyzed cases. We analyzed the association between the HPV prevalence, age at diagnosis, histopathological subtypes, tumor grade, tumor stage, nutrition [albumin and body mass index (BMI)], comorbidity (hypertension and diabetes mellitus), cigarette smoking, and alcohol abuse. The HPV type-specific information included single and multiple infections. Differences in categorical variables were assessed using Pearson's Chi square test or Fisher's exact test. Mann-Kendall test was performed to examine the significance of the HPV and phimosis prevalence trends. Data analyses were performed with SPSS software v.20.0 (IBM Corp., Armonk, NY, USA). Statistical significance was set at two-sided 0.05.

## Results

**Figure 1:**
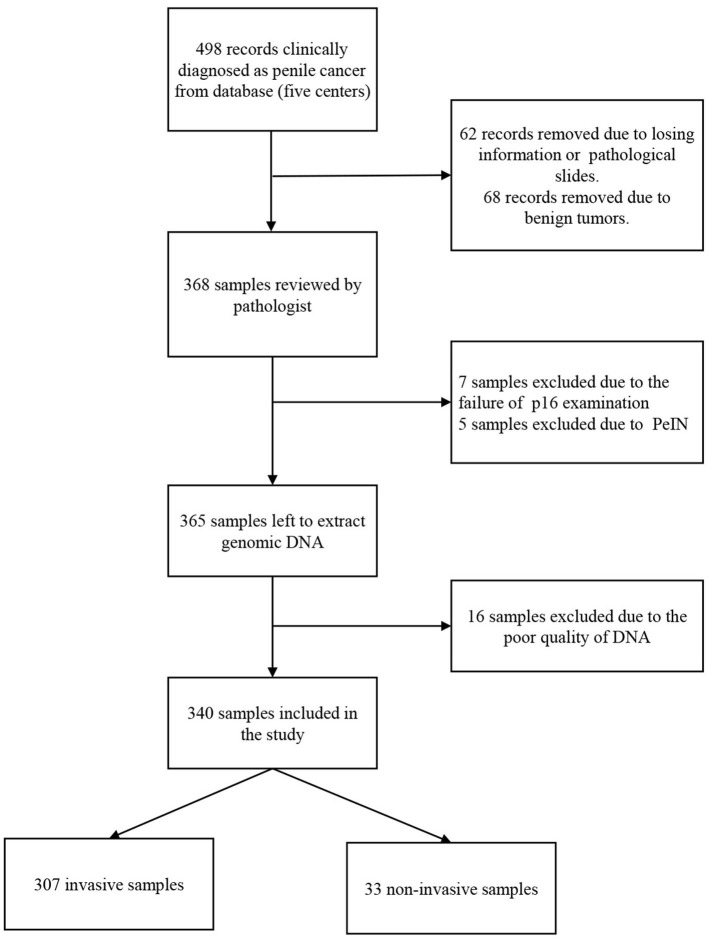
Flow diagram of patient identification and selection.

A total of 340 penile cancers were HPV DNA analyzed and included in the final analyses. Figure 1 shows the algorithm of the study. The HPV DNA prevalence was 48.8% in penile cancers (Table 1), with most HPV infections presenting as high-risk (163/166, 98.2%). HPV16 was the most common type in each histological group (Table 2; 129/166, 77.7%). The second most common type was HPV18 (25/166, 15.1%). HPV16 and 18 accounted for ~88.6% of the HPV positive penile cancers. The positivity of single-type HPV infection was 38.2% (130/340), accounting for 78.3% of the positive population (130/166). The positivity of dual-type infection was 7.1% (24/340), accounting for 14.5% of the positive specimens (24/166). The positivity of triple or more types infection was 3.5% (12/340), accounting for 7.2% of the positive patients (12/166) (Table 2).

**Table 1:** Patient demographics.

**Variables**	**Statistics**
No	340
Age	56 (IQR: 47–65)
BMI	24 (IQR: 21.9–26.2)
HPV infection	166 (48.8%)
Non-invasive cancer	33 (9.7%)
Invasive cancer	307 (90.3%)
**Surgery**	
Glansectomy	22 (6.5%)
Partial penectomy	270 (79.4%)
Radical penectomy	48 (14.1%)
Inguinal lymph node dissection	197 (57.9%)
Pelvic lymphadenectomy	43 (12.6%)
Phimosis	146 (42.9%)
**pT stage**	
Ta	33 (9.7%)
T1	126 (37.1%)
T2	67 (19.7%)
T3	111 (32.6%)
T4	3 (0.9%)
**pN stage**	
N0	226 (66.5%)
N1	47 (13.8%)
N2	35 (10.3%)
N3	32 (9.4%)

**Table 2:** Human papillomavirus (HPV) type-specific relative contribution among HPV DNA-positive penile cancers.

	**Non-invasive penile cancer**		**Invasive penile cancer**	
	**Single**	**Single and Multiple**	**Single**	**Single and Multiple**
**Low risk**				
HPV 6	–	–	–	3
HPV 11	–	–	2	5
HPV 42	–	–	–	4
HPV 43	–	–	1	3
HPV 81	–	–	–	2
**High risk**				
HPV 16	2	2	105	127
HPV 18	–	–	9	25
HPV 31	–	–	2	3
HPV 33	–	–	3	5
HPV 35	–	–	–	1
HPV 39	–	–	–	–
HPV 45	–	–	2	4
HPV 51	–	–	1	1
HPV 52	–	–	–	3
HPV 53	–	–	–	–
HPV 56	–	–	–	4
HPV 58	–	–	–	2
HPV 59	–	–	2	3
HPV 66	–	–	1	2
HPV 68	–	–	–	–
HPV 73	–	–	–	1
HPV 82	–	–	–	–
HPV 83	–	–	–	–

Among penile cancers, the most frequent diagnosis was usual-type squamous cell carcinoma (74.1%). Less frequently, we identified verrucous (7.6%), warty, sarcomatoid, basaloid, and other types (18.3%). HPV DNA was detected in 128 (50.7%) out of 252 cases of usual type, 29 (72.5%) out of 40 cases of warty and basaloid types, 4 (40%) out of 10 cases of sarcomatoid type, and 5 (41.7%) out of 12 cases of histologically rare cases of penile cancer (clear cell, papillary adenosquamous, and pseudohyperplastic tumors). No HPV infection was detected in cancers with verrucous features (Table 3).

**Table 3:** Histologic diagnosis and human papillomavirus DNA prevalence in penile cancers.

	**Non-invasive penile cancers**				**Invasive penile cancers**			
		**HPV DNA positive**		***P***		**HPV DNA positive**		***P***
	***n***	***n***	**%**		***n***	***n***	**%**	
**Histology**								
HPV related	2	2	100	–	40	29	72.5	0.671
Warty					31	22	70.9	
Basaloid	2	2	100		7	5	71.4	
Clear cell					2	2	100	
Non-HPV related	31	0	–	–	267	135	50.6	0.610
Usual	5	0	–		247	128	51.8	
Verrucous	26	0	–		–	–	–	
Sarcomatoid					10	4	40.0	
Papillary					6	2	33.3	
Adenosquamous					3	1	33.3	
Pseudohyperplastic					1	0	–	
LVI				–				0.864
Yes	0	0	–		64	34	53.1	
No	33	2	6.1		243	132	54.3	

No statistically significant differences in HPV DNA prevalence for invasive penile cancers detected among different tumor stages, lymph node stages, or tumor grade (Table 4) The overall HPV prevalence in cases with lymph node metastasis was 45.6%, with a relatively low proportion noted in N1 (40.4%), followed by N3 (46.8%) and N2 (51.4%). Regarding the tumor stage, T2 (44.7%) had a relatively low positivity for HPV DNA compared to T1 (60.3%) and T3-4 (50.9%). For tumor grade, the lowest proportion detected in cases with high differentiation (G1, 52.1%), followed by G3-4 (53.4%), and G2 (54.9%).

**Table 4:** Tumor stage (TNM), tumor grade, and human papillomavirus DNA prevalence in invasive penile cancers.

		**HPV DNA positive**		***P***
	***n***	***n***	**%**	
pT stage				0.059
T1	126	76	60.3	
T2	67	30	44.7	
T3-4	114	58	50.9	
pN stage				0.142
N0	193	112	58.0	
N1	47	19	40.4	
N2	35	18	51.4	
N3	32	15	46.8	
Grade				0.901
G1	142	74	52.1	
G2	122	67	54.9	
G3-4	43	23	53.4	

The HPV infection rates for different age groups were 55.0% (<40 years old), 54.3% (40–50 years old), 59.6% (50–60 years old), 38.1% (60–70 years old), and 34.0% (>70 years old). Thus, penile cancer with HPV infection was more frequently diagnosed in younger patients (*p* = 0.010). No association was observed between HPV type and average age at diagnosis for penile cancers. No statistically significant differences were observed for nutrition status (BMI and albumin), comorbidity (diabetes and hypertension) or lifestyle (cigarette smoking and alcohol drinking) (Table 5).

**Table 5:** Demographics, Smoking, nutrition, comorbidity, and human papillomavirus DNA prevalence in penile cancers.

			**HPV DNA positive**		***P***
		***n***	***n***	**%**	
Age					0.010
<40		40	22	55.0	
40–50		70	38	54.3	
50–60		104	61	59.6	
60–70		76	28	38.1	
≥70		50	17	34.0	
BMI					0.842
<25		214	106	49.5	
≥25		126	61	48.4	
Albumin					0.502
<35		12	7	58.3	
≥35		328	159	48.4	
Heaver smoker					0.073
Yes		127	54	42.5	
No		213	112	52.6	
Diabetes					0.334
Yes		24	14	58.3	
No		316	152	48.1	
Hypertension					0.721
Yes		62	29	46.7	
No		278	137	49.3	
Phimosis					0.144
Yes		146	60	41.1	
No		194	106	56.4	

**Figure 2:**
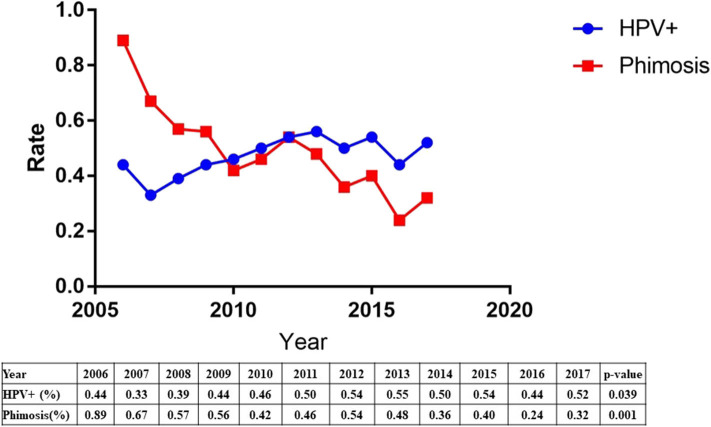
The trend of increasing HPV prevalence and decreasing phimosis from 2006 to 2017.

The etiology of penile squamous cell carcinoma has changed dramatically over the past decade. The data showed a significant increase in HPV prevalence (44–52%, *p* = 0.039) and a significant decrease in phimosis (89–32%, *p* = 0.001) from 2006 to 2017 (Figure 2).

All the specimens were stained to compare p16^*INK4a*^ expression and HPV DNA. The overall percentage of p16^*INK4a*^-positive penile cancer was 45.6%. For non-invasive penile cancers, only two cases (basaloid type) had a both HPV and p16^INK4a^ positivity. HPV-positive invasive penile cancers were found to have a p16^INK4a^ positivity of 82.9% (136/164), while only 17 (17/143, 11.9%) HPV-negative cases were p16^INK4a^ positive. Overall, there was 83.1% (138/166) concordance between the two methods.

## Discussion

Limited epidemiologic data are available for the national incidence of penile cancer in China. We provide reference data for the prevalence of HPV infection and the distribution of HPV genotypes among men with penile cancer. To our knowledge, our data provide the first evidence of a high prevalence of HPV infection in patients with penile cancer in China. Our data indicate that the HPV DNA prevalence (48.8%) in China is as high as that in the United States, European Countries and South America (3, 10). The HPV DNA prevalence presented here for penile cancers is much higher than reported for other Asian countries reported in a recent worldwide study by Laia Alemany (5). The observed differences could be due to the variation of regions and the small number of patients. Although penile cancer is a rare disease, there was a large number of cases of penile cancer in China. Our study indicated that the HPV infection become a more important etiological factor in China nowadays.

HPV prevalence and types have been investigated in fewer studies in Asia compared to Europe, North America and South America. These studies primarily included lesions that were invasive penile cancer and a few cases of intraepithelial neoplasms (11–17) (Supplementary Table 1). However, there was a large degree of heterogeneity in PCR primers and sample number (ranging from 16 to 123).

HPV 16 and HPV 18 were the two most common types, with relative contributions of 76.5 and 15.1%, respectively. The combination of HPV types 16 and 18 greatly increased the relative contribution of HPV to penile cancer to 88.6%. Together, the HPV types 16, 18 and 33 further increased the relative contribution to 91.0%, and the results of this study strongly support the restricted genotype contribution in the pathogenesis of penile cancer in China. Our data also confirm the dominant roles of HPV16 and 18 in all types of penile cancers. According to our data, the 2-valent HPV vaccine, approved by the Chinese Food and Drug Administration, indicated a combined prevalence among HPV DNA-positive cases of 88.6% for penile cancers. Recently, the new 9-valent HPV vaccine has been approved, and 95.2% of patients might receive protection from penile cancer.

It has been reported that HPV-related tumors are diagnosed in younger patients than non-HPV-related cancers, as has been reported for female genitalia cancers and head and neck cancers (7). Our findings also indicated that age might be an important factor related to high-risk HPV infection in penile cancers, which is in agreement with previous studies. Previous studies showed that the age at diagnosis was much younger for women with invasive cervical cancers with specific HPV types, such as HPV 45 (7). However, we could not identify these significant associations, with the possible explanation of the majority of patients with positive HPV 16 infection.

The etiology of penile squamous cell carcinoma has shifted over the past decade. Our results indicated a significant increase in HPV prevalence and a significant decrease in phimosis. One explanation may be related to increase awareness of early detection of phimosis and prevention of phimosis. The other explanation was the sharp increased population floating in the past decade in China, and HPV are more likely to spread.

Our study indicated that HPV DNA may be associated with aggressive tumors, which was similar with a previous report (18). HPV DNA prevalence tends to be associated with high-grade tumors (HPV prevalence 53.4% for G3-4 vs. 43.4% for G1). However, no statistically significant differences were detected among different tumor, lymph node stages, or tumor grade. Our study did not analyze the association between the prognosis and HPV prevalence, because the median follow-up time was too short. A recent published international multicenter study including 230 patients in our study found HPV status could separate the prognosis of pN2-3 patients, and pN2-3 high risk HPV negative group was associated with only 32% 5-year survival (19).

The success of HPV testing in clinical practice is largely dependent on its high sensitivity and negative predictive value. A previous study showed that the PCR-RDB HPV test is a reliable, sensitive, and accurate method for cervical cancer screening (8, 20). In addition, to avoid false-negative results, an internal quality control was used with the PCR-RDB method to evaluate the occurrence of false negatives. Our results also indicated a good concordance rate between the PCR-RDB HPV test and immunohistochemistry for p16^*INK4a*^ expression, which further confirms the PCR-RDB HPV test as a reliable and easy-to-use detection method. This concordance is in agreement with a previous global study (5).

We adopted the new 2016 WHO classification and applied it to our large cohort. The updated subtypes and grades were applied to 340 cases. We confirmed the HPV differences between the two general groups and found grade discrepancies between pathological subtypes. The connection between subtype and grade may indicate a corresponding HPV positivity rate and warrants further research to determine an individualized treatment approach.

To our knowledge, our study is the first large multicenter examination of HPV prevalence and the distribution of HPV genotypes in China. The strengths of the study include the use of the same protocol for specimen collection, central pathological review, and classification by an experienced pathologist, and well-standardized virus detection in a single central laboratory using two different markers of viral presence at the levels of viral DNA and p16^*INK4a*^ ed information on HPV positivity according to the recent WHO update of pathological subtypes and grades. This study, however, has some potential limitations. The included cases were obtained from five level three tertiary hospitals, mainly on the eastern coast of China, which may represent the developed region in China. Thus, bias could arise due to unbalanced regional development.

In conclusion, our study provided information regarding HPV infection in penile cancers in China using robust methods. Approximately half of the penile cancer cases were related to HPV infection, as detected with a PCR-RDB HPV test. As demonstrated in our study population, genotypes 16, 18, and 33 are the predominant HPV types in invasive penile cancer. Our findings highlight the phimosis related penile cancers have been declining. Regional differences in HPV genotype distribution and the carcinogenic impact of HPV indicate the need to be mindful of HPV control and prevention.

## Data Availability Statement

The datasets generated for this study are available on request to the corresponding author.

## Ethics Statement

The studies involving human participants were reviewed and approved by the Institutional Review Board of Fudan University Shanghai Cancer Center, The Affiliated Hospital of Qingdao University, Hainan General Hospital, Northern Jiangsu People's Hospital, and Affiliated Hospital of Jiangnan University. The patients/participants provided their written informed consent to participate in this study.

## Author Contributions

DY, YZ, and WG designed research and wrote the manuscript. PZ reviewed the pathology slides. BW, SJ, and YW performed the HPV DNA genotype analysis and immunohistochemical analysis. GZ, JZ, XD, and QW performed data collection and processing of data. All authors approved the manuscript.

## Conflict of Interest

The authors declare that the research was conducted in the absence of any commercial or financial relationships that could be construed as a potential conflict of interest.
