# Supplementary material for: Importance of HPV in Chinese Penile Cancer: A Contemporary Multicenter Study
Source: Front Oncol. 2020 Sep 4;10:1521. doi: 10.3389/fonc.2020.01521 (PMC7498546; doi:10.3389/fonc.2020.01521)
Supplement: Supplementary file 1 [file Table_1.DOCX]

**Supplementary table 1**

**Compare the HPV prevalence of China versus other Asian regions**

| Region | Publication year | Method of detection | | Sample | n | % |
| --- | --- | --- | --- | --- | --- | --- |
| Japan(1) | 1993 | Specific primers  (HPV 16 18 33) | FFPE | | 123 | 58.5% |
| Hong Kong(2) | 1994 | Specific primers  (HPV 16 18) | FFPE | | 41 | 14.6% |
| Japan(3) | 1994 | Mixed primers  (9 types) | FFPE | | 13 | 53.8% |
| Thailand(4) | 2006 | Consensus primers  (43 types) | FFPE | | 65 | 81.5% |
| Japan(5) | 2008 | Consensus primers  (9 types) | FFPE | | 25 | 11.5% |
| Japan(6) | 2010 | Consensus primers  (43 types) | FFPE | | 16 | 75.0% |
| Vietnam(7) | 2013 | Consensus primers  (43 types) | FFPE | | 120 | 22.5% |
| Myanmar(8) | 2016 | Consensus primers  (7 types) | FFPE | | 30 | 26.7% |
| China  (This study) | 2020 | Consensus primers  (23 types) | FFPE | | 340 | 48.8% |

1. Iwasawa A, Kumamoto Y, Fujinaga K. Detection of human papillomavirus deoxyribonucleic acid in penile carcinoma by polymerase chain reaction and in situ hybridization. J Urol. 1993;149(1):59-63.

2. Chan KW, Lam KY, Chan AC, Lau P, Srivastava G. Prevalence of human papillomavirus types 16 and 18 in penile carcinoma: a study of 41 cases using PCR. J Clin Pathol. 1994;47(9):823-6.

3. Suzuki H, Sato N, Kodama T, Okano T, Isaka S, Shirasawa H, et al. Detection of human papillomavirus DNA and state of p53 gene in Japanese penile cancer. Jpn J Clin Oncol. 1994;24(1):1-6.

4. Senba M, Kumatori A, Fujita S, Jutavijittum P, Yousukh A, Moriuchi T, et al. The prevalence of human papillomavirus genotypes in penile cancers from northern Thailand. J Med Virol. 2006;78(10):1341-6.

5. Yanagawa N, Osakabe M, Hayashi M, Tamura G, Motoyama T. Detection of HPV-DNA, p53 alterations, and methylation in penile squamous cell carcinoma in Japanese men. Pathol Int. 2008;58(8):477-82.

6. Senba M, Mori N, Wada A, Fujita S, Yasunami M, Irie S, et al. Human papillomavirus genotypes in penile cancers from Japanese patients and HPV-induced NF-kappaB activation. Oncol Lett. 2010;1(2):267-72.

7. Do HT, Koriyama C, Khan NA, Higashi M, Kato T, Le NT, et al. The etiologic role of human papillomavirus in penile cancers: a study in Vietnam. Br J Cancer. 2013;108(1):229-33.

8. Mu Mu S, Hlaing Myat T, Khin Saw A, Aye Aye M, Mya T, Khin Shwe M, et al. Determination of Oncogenic Human Papillomavirus (HPV) Genotypes in Anogenital Cancers in Myanmar. Acta Med Okayama. 2016;70(2):103-10.
